# Cadmium Accumulation Characteristics in Turnip Landraces from China and Assessment of Their Phytoremediation Potential for Contaminated Soils

**DOI:** 10.3389/fpls.2016.01862

**Published:** 2016-12-09

**Authors:** Xiong Li, Xiaoming Zhang, Ya Yang, Boqun Li, Yuansheng Wu, Hang Sun, Yongping Yang

**Affiliations:** ^1^Key Laboratory for Plant Diversity and Biogeography of East Asia, Kunming Institute of Botany, Chinese Academy of SciencesKunming, China; ^2^Germplasm Bank of Wild Species, Kunming Institute of Botany, Chinese Academy of SciencesKunming, China; ^3^University of Chinese Academy of SciencesBeijing, China; ^4^College of Plant Protection, Yunnan Agricultural UniversityKunming, China

**Keywords:** turnip, cadmium, soil pollution, phytoremediation, hyperaccumulator

## Abstract

Heavy metal (HM) pollution is a global environmental problem that threatens ecosystem and human health. Cadmium (Cd) pollution is the most prominent HM pollution type because of its high toxicity, strong migration, and the large polluted area globally. Phytoremediation of contaminated soil is frequently practiced because of its cost-effectiveness and operability and because it has no associated secondary pollution. High-accumulation plants, including those identified as hyperaccumulators, play an important role in phytoremediation. Therefore, screening of plants to identify hyperaccumulators is important for continued phytoremediation. In the present study, we investigated the Cd tolerance and accumulation capabilities of 18 turnip landraces from China under a soil experiment with known Cd level. The results indicated that turnip has a high capacity for Cd accumulation. Furthermore, significant differences in Cd tolerance and accumulation characteristics were found among different landraces when they grew at 50 mg kg^-1^ (dry weight) Cd concentration. Among the studied landraces, five turnip landraces met the requirements of Cd hyperaccumulators and three landraces were identified as potential candidates. However, the total Cd content accumulated by individual plant of different turnip landraces was dependent on both the Cd accumulation capacity and plant biomass. Compared with some reported Cd hyperaccumulators, turnip not only shows a high Cd-accumulation capacity but also has rapid growth and a wide distribution area. These advantages indicate that turnip may have considerable potential for phytoremediation of Cd-contaminated soil. Furthermore, the study also indicates that it is not advisable to consume turnip cultivated in an environment that exceeds safe Cd levels.

## Introduction

Heavy metal (HM) pollution is a global environmental problem, which seriously threatens ecosystem safety, agricultural production and human health (Gao, 2016). Cadmium (Cd) contamination has become the most prominent soil HM pollution issue as a result of its high toxicity, strong migration, and large pollution area globally (Moreno-Caselles et al., 2000; Wei et al., 2006). Soil Cd has three main sources including atmospheric deposition, irrigation with sewage and use of pesticides and chemical fertilizers (Sikka et al., 2009). Enrichment of Cd in soil can change soil physical and chemical properties, reduce the richness, diversity, and activity of soil microorganisms, and inhibit soil respiration, subsequently affecting soil fertility (Zheng and Shang, 2006). Because Cd is a non-essential element in plants, excessive Cd absorbed in plants directly influences their normal physiological function. The symptoms are usually characterized by growth inhibition, leaf chlorosis, metabolic block, and even plant death (Abd Allah et al., 2015; Hashem et al., 2016). Moreover, the absorbed Cd can poison other organisms, including humans, along the food chain (Amna et al., 2015; Tauqeer et al., 2016). After entering human body, Cd is selectively enriched in the kidney and liver, damaging organ functions (Wu, 2015). Furthermore, Cd can also produce toxicity to other systems of human body, and even induce cell distortion and cancerization (Wu, 2015). One of the most famous accidents was the “Itai-Itai Disease” that occurred in Toyama Prefecture of Japan in 1960s (Baba et al., 2013). Several events of human Cd poisoning resulting from soil Cd pollution have subsequently been reported around the world (Sterckeman et al., 2000; Du et al., 2013).

With the frequent occurrence of HM pollution incidents in recent years, increasing attention has been paid to the prevention and remediation of HM pollution. The remediation of HM-contaminated soil refers to using physical, chemical, biological, or combined methods to transfer or absorb HMs and reduce their concentrations to below harmful levels (Buendia-Gonzalez et al., 2010). The main physical and chemical remediation technologies are curing stability, leaching, chemical oxidation–reduction and soil electrokinetic remediation (Wu, 2015; Zhang, 2015). Although these methods can bring rapid effects, most of them are costly, produce secondary pollution and can damage soil properties (Luo et al., 2008). Therefore, these physicochemical methods are difficult to apply widely. In contrast, biological remediation of contaminated soil involves decreasing the HM concentrations in the soil through metabolic activities of organisms, and is mainly conducted through microbial remediation and phytoremediation (Xiao et al., 2013). Microbial remediation is typically achieved through biological oxidation–reduction and biological adsorption, and has the advantages of low cost, eco-friendly effect and operation etc. (Xiao et al., 2013). However, microbial remediation alone is also associated with problems such as the strong environmental effect on microbial growth and the inherent difficulties in collecting bacteria (Wu, 2015). Therefore, phytoremediation of polluted soil has become the predominant option in recent years (Ehsan et al., 2014; Kamran et al., 2014; Amna et al., 2015).

Phytoremediation technology is dependent on plant adsorption and absorption characteristics for HMs (Adiloglu et al., 2016; Bokhari et al., 2016). As plants have diverse tolerance and accumulation abilities for the various HMs, plants that can tolerate or accumulate higher HM concentrations are defined as hyperaccumulators (Kramer, 2010). For Cd hyperaccumulators, the threshold value of Cd concentration is 100 mg kg^-1^ in the dried AG part (Baker and Walker, 1990). In recent years, the screening and application of Cd hyperaccumulators has been intensively conducted worldwide, and increasing numbers of Cd hyperaccumulators have been identified and gradually applied in the remediation of Cd contaminated soil (Xiao et al., 2013). Although hyperaccumulators have a strong accumulation capacity for Cd, many of them usually grow slowly and have a low biomass, which greatly limits their remediation efficiency (Xiao et al., 2013). Therefore, screening further plant species with a high potential for Cd accumulation, combined with molecular biology methods to improve the growth and enrichment ability of hyperaccumulators or common plants, is an effective approach to improving the phytoremediation efficiency of contaminated soil.

Brassicaceae is a relatively large family of angiosperms, which includes about 360 genera and 3,700 species across the world. Many cruciferous plants are common vegetables and oilseed crops. These plants are closely related to soil safety and human health. Cruciferous plants are generally considered to represent the largest proportion of HM hyperaccumulators (He et al., 2009), and the earliest identified and most reported Cd hyperaccumulators are members of the Brassicaceae family (Zhao et al., 2002). In recent years, many studies of Cd absorption and accumulation have been performed for common cruciferous crops, such as rape (Carrier et al., 2003; Wang et al., 2009), Chinese cabbage (Aziz et al., 2015; Wu et al., 2015), pakchoi (Chen et al., 2010; Zhou et al., 2016), broccoli (Pedrero et al., 2008), and radish (Lagerwer, 1971; Vitoria et al., 2003; Lin et al., 2014). These studies provide useful information on food safety and potential of soil remediation of these plants. Turnip (*Brassica rapa* var. *rapa*), a cruciferous biennial plant, has been widely cultivated in Europe, Asia and America for hundreds of years as a vegetable or fodder. As early as the 1970s, scientists in America began to examine the Cd absorption and accumulation in turnip (Page et al., 1972; Bingham et al., 1975). Page et al. (1972) found that the Cd concentration in leaves of turnip could reach 469 μg g^-1^ tissues when the plants were cultured in complete nutrient solution with a Cd concentration of 1.0 μg mL^-1^. Bingham et al. (1975) reported that turnip greens (tops) could contain up to 354 μg g^-1^ Cd when growing on soil treated with 160 μg g^-1^ Cd. These studies indicated that turnip was a high-Cd-accumulation plant as Arthur et al. (2000) divided. Thereafter, several studies about turnip accumulating Cd were performed in Asian countries (Mani and Kumar, 2004; Mani et al., 2007; Molahoseini and Feizi, 2012; Parveen et al., 2013). However, the Cd concentrations reported in these studies were generally very low. The results of two studies in India showed that the highest Cd accumulations were just 3.23 mg kg^-1^ in turnip roots and 2.13 or 2.17 mg kg^-1^ in shoots, respectively (Mani and Kumar, 2004; Mani et al., 2007). A similar low Cd accumulation in turnip was also reported in another recent Indian study, in which the highest Cd concentration was less than 15 mg kg^-1^ in roots and even lower in leaves when the plants were irrigated by treated municipal wastewater (Parveen et al., 2013). In addition, a study performed in Iran showed that minimal enrichment of Cd in turnip grown under wastewater irrigation (Molahoseini and Feizi, 2012). These previous studies indicated that the Cd absorption and accumulation ability varies considerably among turnip plants from different regions (e.g., America and Asia), which might be because the plants belonged to different genotypes. China is a major planting country of turnip and has highly abundant turnip germplasm resources. However, systematic studies of the tolerance, absorption and accumulation of turnip of Cd or other HMs remain absent in China. Because of the lack of experimental data, the Cd accumulation within turnip is currently poorly defined in terms of food safety. Furthermore, few studies have considered the potential value of turnip in phytoremediation of Cd-contaminated soil. In the present study, we analyzed the tolerance of turnip to Cd stress and Cd accumulation differences of multiple turnip landraces. The results could provide a theoretical basis for further assessing the food safety risk and the remediation potential of Cd-contaminated soil under turnip.

## Materials and Methods

### Screening Test for Cd Concentration

We collected as many different turnip landraces as possible from the main planting areas in China and then numbered them. From these, the landrace “KTRG-B19” from Mianning Country, Sichuan Province of China was randomly selected to investigate the tolerance ability to Cd stresses. The seeds were sown in a seedling-raising plate under natural conditions with appropriate watering. When the seedlings grew to the trefoil stage, they were transplanted to uniform watertight pots (length: 30 cm; width: 20 cm; height: 15 cm) in the greenhouse (12-h light/12-h darkness, 22°C, 50–60% relative humidity). The humus soil was used. Each pot was planted with nine seedlings with consistent growth.

After a 2-week recovery period, the plants were treated with CdCl_2_ solution. In order to select an appropriate Cd concentration for the later material treatment, we set a soil Cd^2+^ concentration gradient of 0, 1.22, 3.06, 6.12, 12.24, 30.60, and 61.20 mg kg^-1^ (DW), respectively. After treatment for 3 weeks, the plant phenotype was recorded and the AG part of the three plants for Cd^2+^ treatment concentrations of 1.22, 6.12, 30.60, and 61.20 mg kg^-1^ was collected to measure the Cd content.

### Material Preparation and Treatment

According to the results of above Cd treatment, we selected the concentration of 50 mg kg^-1^ Cd^2+^ in the mucky soil (DW) to explore the Cd accumulation differences among different landraces. A total of 18 turnip landraces from different regions were used for the experiments (**Table 1**). These landraces showed clear differences in morphological characteristics (Supplementary Figure S1), which might indicate various genetic resources. Two uniform watertight boxes (length: 64 cm; width: 44 cm; height: 26 cm) were prepared for each landrace. One box was set as the control group and the other was used for the treatment group. We used CdCl_2_ to provide Cd^2+^ and it was mixed thoroughly with the soil. Each box was equipped with 15 kg of dried soil.

**Table 1:** Seed origins and local names of different turnip landraces.

Landraces	Origins	Local names
KTRG-B03	Nangqên county, Qinghai, China	Yuankanin
KTRG-B06	Chindu county, Qinghai, China	Yuankanin
KTRG-B13	Weixi county, Yunnan, China	Manjing
KTRG-B14	Xiangcheng county, Sichuan, China	Yuankanin
KTRG-B16	Lijiang city, Yunnan, China	Manjing
KTRG-B19	Mianning county, Sichuan, China	Manjing
KTRG-B22	Eryuan county, Yunnan, China	Manjing
KTRG-B25	Jianchuan county, Yunnan, China	Manjing
KTRG-B28	Yunlong county, Yunnan, China	Manjing
KTRG-B31	Jianchuan county, Yunnan, China	Manjing
KTRG-B36	Lanping county, Yunnan, China	Manjing
KTRG-B45c	Lanping county, Yunnan, China	Manjing
KTRG-B48a	Shangri-La county, Yunnan, China	Manjing
KTRG-B48b	Shangri-La county, Yunnan, China	Manjing
KTRG-B50	Shouguang city, Shangdong, China	Manjing
KTRG-B54	Ninglang county, Yunnan, China	Yuangen
KTRG-B56	Changji city, Xinjiang, China	Qiamagu
KTRG-B57	Qüxü county, Tibet, China	Newma

Seeds of each turnip landrace were regularly sown in nine dots (3 × 3) in both the control and treatment boxes. The boxes were placed under natural light and temperature, with appropriate watering. After seedling emergence, a total of nine seedlings with consistent growth remained (one seedling at each dot). After growing for a month, the AG and UG parts of the plants were collected, respectively, and the roots were cleaned using ultrapure water. The samples were dried under 80°C for 48 h and their weights were recorded. The AG and UG parts of three plants of each landrace were then used to measure the Cd content.

### Cd Concentration Measurement

To draw the standard curve of Cd content, the Cd standard solution (1 mg mL^-1^) was diluted with 5% HNO_3_ to 10 mg L^-1^ stock solution. The stock solution was then prepared into 0, 0.1, 0.2, 0.4, and 1 mg L^-1^ standard solutions and mixed for detection. The solutions were detected by an Inductively Coupled Plasma Spectrometer (ICP-OES optima 8000, Perkin Elmer, US) using the wavelength of 214.44 nm. The standard curve was drawn only when the linear correlation coefficient was greater than 0.99.

Approximately 0.2–1.0 g dried samples were added to polytetrafluoroethylene digestion tanks, then, 5 mL HNO_3_ was injected and the tanks were left to stand. When the reactions finished, the tanks were sealed with caps and put into a microwave digestion instrument (WX-8000) using the following digestion procedure: 100°C, 3 min; 140°C, 3 min; 160°C, 3 min; 180°C, 3 min; 190°C, 15 min. When the temperature cooled below 50°C, the digestion tanks were taken to the fume hood. The digestion solutions were transferred to 50 mL volumetric flasks and fixed volume to 50 mL by rinsing three or four times using ultrapure water. The blank control was treated using the same method. The sample solutions were detected under the same wavelength and the Cd contents were calculated according to the standard curve.

### Statistical Analysis

Statistical analyses were performed using SPSS version 18.0. One-way ANOVA was used to analyze significant differences among multiple samples and an independent-samples *t*-test was used between each pair of samples. All significant differences were identified at 0.05 levels. Linear regression analysis was used to identify the correlations.

## Results

### Effects of Cd at Different Concentrations on Turnip

**FIGURE 1:**
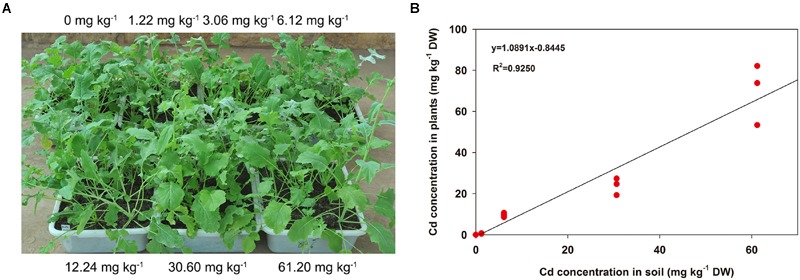
**Morphological differences and Cd accumulation changes in AG part of turnip plants under different Cd concentrations. (A)** Morphological differences of turnip plants treated with different Cd concentrations. **(B)** Linear regression analysis between Cd accumulation in the AG part of turnip plants and the Cd concentrations in soil.

When treated by Cd at different concentrations for 3 weeks, the morphology of turnip plants showed no significant differences compared with the control materials (**Figure 1A**). As the Cd concentration added to the soil increased, the Cd content accumulated in the AG part of plants was improved (**Figure 1B**). Furthermore, these two indexes showed a significant linear relationship (*F* = 160.3184, *P* < 0.001; **Figure 1B**).

### Effects of Cd Treatment on Different Turnip Landraces

**FIGURE 2:**
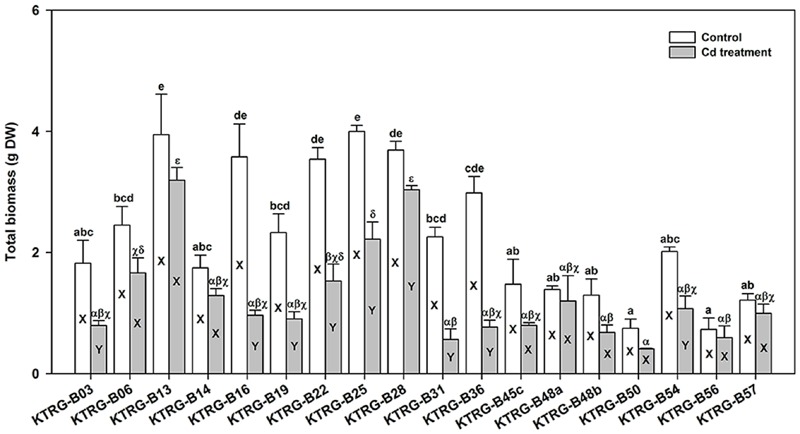
**The total biomass (g DW) differences of different turnip landraces under control and Cd treatment conditions.** Data represent means ± SE. Bars labeled with different letters are significantly different (*n* = 3, *P* < 0.05) between control and Cd treatment samples (X and Y) or among different landraces (a–e or α–𝜀).

Because turnip plants could tolerate at least 61.20 mg kg^-1^ Cd^2+^ in soil (Section Screening Test for Cd Concentration), we selected the soil concentration of 50 mg kg^-1^ Cd^2+^ to investigate the tolerance and accumulation abilities of different turnip landraces. When growing for the same time after sowing (a month), we found that the 18 turnip landraces showed substantial differences in plant growth under the control condition (**Figure 2**). The average total biomass ranged from 0.73 (KTRG-B56) to 4.00 g (KTRG-B25). When treated by Cd, the biomasses of all 18 landraces decreased by diverse degrees compared with their respective control materials (**Figure 2**) and thus the mean total biomass of plants in the Cd-treated soil also varied between 0.41 (KTRG-B50) and 3.19 g (KTRG-B13; **Figure 2**). Among all landraces, the growth of nine landraces (KTRG-B03, KTRG-B16, KTRG-B19, KTRG-B22, KTRG-B25, KTRG-B28, KTRG-B31, KTRG-B36 and KTRG-B54) was significantly inhibited by Cd (**Figure 2**), whereas the remaining landraces (KTRG-B06, KTRG-B13, KTRG-B14, KTRG-B45c, KTRG-B48a, KTRG-B48b, KTRG-B50, KTRG-B56 and KTRG-B57) showed a stronger tolerance to the Cd concentration used in this study (**Figure 2**).

### Cd Accumulation Differences among Turnip Landraces

**FIGURE 3:**
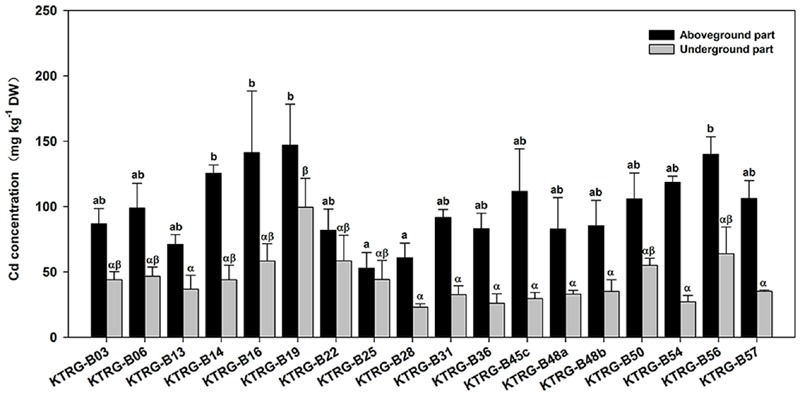
**Cd concentrations (mg kg^-1^) in the AG and UG parts of different turnip landraces.** Data represent means ± SE. Bars labeled with different letters are significantly different (*n* = 3, *P* < 0.05) among different landraces (a, b or α, β).

The Cd accumulation concentrations in both the AG and UG parts were different among the investigated landraces (**Figure 3**). The average Cd concentrations in the AG part ranged from 52.94 (KTRG-B25) to 146.95 mg kg^-1^ DW (KTRG-B19) with a mean value of 99.48 mg kg^-1^ DW (**Figure 3**), which were greater than the concentration in the soil. Specially, Cd accumulation exceed 100 mg kg^-1^ in the leaves of KTRG-B14 (125.27 mg kg^-1^), KTRG-B16 (141.25 mg kg^-1^), KTRG-B19 (146.95 mg kg^-1^), KTRG-B45c (116.63 mg kg^-1^), KTRG-B50 (105.88 mg kg^-1^), KTRG-B54 (118.47 mg kg^-1^), KTRG-B56 (139.87 mg kg^-1^) and KTRG-B57 (106.27 mg kg^-1^), whereas most of the others were very close to this value (**Figure 3**). The average Cd concentrations in UG part ranged from 8.17 (KTRG-B54) to 81.52 mg kg^-1^ DW (KTRG-B16; **Figure 3**) and the mean value was 44.03 mg kg^-1^ DW (**Figure 3**). The scope of this variation was much greater than that of the AG part (**Figure 3**). For each turnip, the Cd concentration in the AG part was higher than that in the UG part (**Figure 3**). To understand the absorption and transportation characteristics, two parameters including EC and TF were introduced. In the present study, the ECs of AG part of all landraces were greater than 1 (**Table 1**), and the mean value was 1.99. In detail, KTRG-B16 (2.83), KTRG-B19 (2.84) and KTRG-B56 (2.80) showed high enrichment ability whereas KTRG-B13 (1.42), KTRG-B25 (1.06) and KTRG-B28 (1.22) had low ECs (**Table 1**). Similarly, the TFs of all landraces were also greater than 1 (**Table 1**), with values that ranged from 1.36 (KTRG-B25) to 4.82 (KTRG-B54) with a mean value of 2.61 (**Table 1**).

**FIGURE 4:**
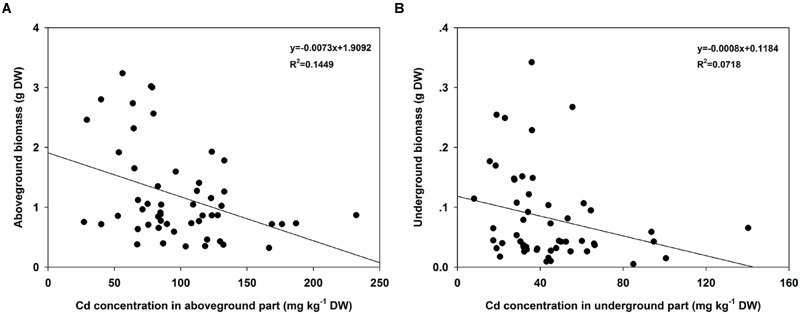
**Regression analyses between biomasses and Cd concentrations among different turnip landraces. (A)** Regression analysis of biomasses of AG parts and corresponding Cd concentrations among different turnip landraces. **(B)** Regression analysis of biomasses of UG parts and corresponding Cd concentrations among different turnip landraces.

**FIGURE 5:**
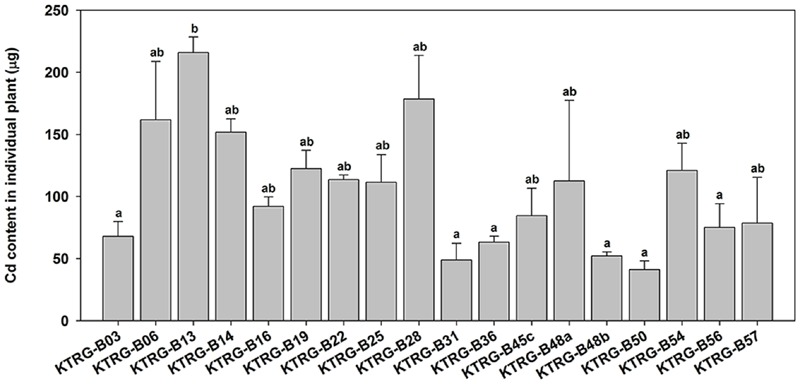
**Estimated total Cd contents (μg) accumulated by individual plants of different turnip landraces.** Data represent means ± SE. Bars labeled with different letters are significantly different (*n* = 3, *P* < 0.05) among different landraces.

Regression analyses indicated that the Cd concentrations in the AG part of turnip plants were significantly correlated with the corresponding biomasses (*F* = 8.6456, *P* = 0.0049; **Figure 4A**). However, the correlations in UG part were not significant (*F* = 3.9475, *P* = 0.0523; **Figure 4B**). To further assess the Cd accumulation effects of different turnip landraces, we estimated the total Cd content accumulated by individual plant using the following formula: C_T_= C_AG_×B_AG_+C_UG_×B_UG_ where C_T_ is the total Cd content (μg); C_AG_ and C_UG_ is the Cd concentration in the AG and UG parts (μg g^-1^), respectively; and B_AG_ and B_UG_ is the biomass of the AG and UG parts (g), respectively. The results showed that total Cd content of the single plant was different from each other among 18 landraces (**Figure 5**). Individual plants could enrich a minimum of 41.08 (KTRG-B50) and maximum of 215.92 μg (KTRG-B13) Cd element (**Figure 5**), and the mean accumulation content was 105.17 μg.

## Discussion

### Turnip is a High Cd Accumulator

Significant interspecific differences in plant absorption and accumulation of HMs have been demonstrated. Arthur et al. (2000) divided plants into three categories based on their HM absorption and accumulation capabilities: high accumulation type, moderate accumulation type and low accumulation type. Turnip has previously been described as belonging to high Cd accumulation plants (Arthur et al., 2000); however, supporting data and evidence were limited. According to a previous survey, the mean Cd concentration in soil around the world was about 0.35 mg kg^-1^ (He et al., 1998), and the Cd concentration in normal soil usually did not exceed 1 mg kg^-1^. In this study, a Cd concentration gradient from 1.22 to 61.20 mg kg^-1^ was used to treat turnip plants, which could represent mildly, moderately and severely contaminated soil. The unscathed morphology compared with the control samples indicated that turnip plants had very strong tolerance to Cd stress. Furthermore, Cd content absorbed by turnip increased with the increasing Cd concentration in soil. At the highest Cd treatment concentration, Cd concentration in turnip plants (69.80 mg kg^-1^) was close to the critical value of Cd hyperaccumulators (Baker and Brooks, 1989). Our results indicated that this turnip landrace investigated (KTRG-B19) had strong accumulation ability for soil Cd^2+^.

We next examined a total of 18 turnip landraces to further confirm their absorption capacities. The Cd concentrations accumulated in AG parts of all turnip landraces were more than 50 mg kg^-1^, and those of eight landraces exceeded 100 mg kg^-1^ (**Figure 3**). The EC value is usually used to reflect the ability of plants to accumulate environmental pollutants, which is indicated as the ratio of pollutant concentration in plants to that in the environment (He, 2013). In the present study, Cd ECs of AG parts of all turnip landraces were greater than 1 (**Table 2**). In addition, another notable finding of our study was that the accumulation time was within a month, which indicated that Cd absorption and accumulation of turnip might be further enhanced as the plant continues to grow. Previous studies provide evidence to support this speculation; for example, Niu (2012) reported that Cd accumulation concentrations in AG parts of six rape landraces gradually increased from the seedling stage to maturity stage. In addition, Chen (2013) found that Cd concentration in *Bidens pilosa* roots increased during the 30- to150-day life stage under various Cd treatment concentrations.

**Table 2:** Cd ECs and TFs of different turnip landraces.

Landraces	Enrichment coefficients	Translocation factors
KTRG-B03	1.73 ± 0.23ab	2.04 ± 0.35αβ
KTRG-B06	1.98 ± 0.38ab	2.28 ± 0.58αβ
KTRG-B13	1.42 ± 0.15ab	2.44 ± 0.90αβ
KTRG-B14	2.51 ± 0.13ab	3.16 ± 0.66αβ
KTRG-B16	2.83 ± 0.94b	2.33 ± 0.33αβ
KTRG-B19	2.94 ± 0.62b	1.49 ± 0.16α
KTRG-B22	1.64 ± 0.32ab	1.63 ± 0.38αβ
KTRG-B25	1.06 ± 0.24a	1.36 ± 0.26α
KTRG-B28	1.22 ± 0.22ab	2.69 ± 0.50αβ
KTRG-B31	1.84 ± 0.12ab	3.04 ± 0.59αβ
KTRG-B36	1.66 ± 0.24ab	3.31 ± 0.46αβ
KTRG-B45c	2.24 ± 0.65ab	3.69 ± 0.57αβ
KTRG-B48a	1.66 ± 0.48ab	2.59 ± 0.90αβ
KTRG-B48b	1.71 ± 0.39ab	2.56 ± 0.36αβ
KTRG-B50	2.12 ± 0.40ab	1.94 ± 0.37αβ
KTRG-B54	2.37 ± 0.09ab	4.82 ± 1.27β
KTRG-B56	2.80 ± 0.27b	2.65 ± 0.82αβ
KTRG-B57	2.13 ± 0.27ab	3.00 ± 0.31αβ

*Data represent means ± SE. Data of each index labeled with different letters are significantly different (*n* = 3, *P* < 0.05) among different landraces (a, b or α, β).*

Overall, our results have clearly shown that turnip has a high Cd accumulation ability, which supports the conclusion of Arthur et al. (2000). However, the maximum value of Cd accumulation in turnip still requires further study. In addition, whether turnip has strong ability in absorbing other HMs is unclear and also requires additional research.

### Intraspecific Variation of Cd Tolerance and Absorption in Turnip

A noticeable intraspecific variation in plant accumulation of Cd has also been reported. High and low Cd accumulation cultivars have been identified in many crops, such as rape (Niu, 2012; Wu et al., 2015), pakchoi (Zhou et al., 2016), watercress (Wang et al., 2015), and Chinese leaf mustard (Dai et al., 2012). Differences in Cd absorption and accumulation among different turnip cultivars or genotypes also have been indicated in studies around the world (Bingham et al., 1975; Molahoseini and Feizi, 2012; Parveen et al., 2013). The results of the present study further demonstrated that there are intraspecific differences of Cd tolerance and accumulation in turnip. **Figure 2** shows that plant growth varied significantly among different turnip landraces under normal conditions. Under the same Cd conditions, plant growth of different turnip landraces was inhibited to various degrees (**Figure 2**). The results suggested that these turnip landraces had diverse tolerances to Cd stress and different Cd accumulation abilities. The maximum Cd concentration (KTRG-B19) in the AG part was 2.78 times the minimum value (KTRG-B25) while the ratio of the highest Cd concentration (KTRG-B19) and the lowest value (KTRG-B28) was 4.33 in the UG part. Interestingly, we found that the AG biomass was significantly negatively correlated with the corresponding Cd concentration (**Figure 4A**). This finding indicates that inhibited plant growth of some turnip landraces might result from large absorption of Cd. However, this was not observed in the UG part (**Figure 4A**), which could be attributed to the large TFs that represented a strong transfer capability of Cd from the plant UG part to the AG part (He, 2013).

As a vegetable plant, intraspecific differences of Cd accumulation might be of great relevance to human life. On the one hand, for food safety, cultivars with low capacity of Cd accumulation or Cd pollution-safe cultivars are an economical and effective strategy to restrict Cd transfer into the food chain. On the other hand, cultivars with high capacity of Cd accumulation are excellent candidates for the phytoremediation of contaminated soil. For turnip, all the landraces measured in the present study belong to the high Cd accumulation type. This suggests that it is not advisable to consume turnips cultivated in environments where safe Cd levels are exceeded. Moreover, based on the different results from different countries (Page et al., 1972; Bingham et al., 1975; Mani and Kumar, 2004; Mani et al., 2007; Molahoseini and Feizi, 2012; Parveen et al., 2013), we should consider comparing different turnip cultivars or genotypes worldwide and screening for low and high-Cd-accumulating cultivars. As mentioned before, the large intraspecific differences of Cd accumulation in turnip might be caused by various genetic backgrounds. However, the environmental Cd concentrations also influenced the Cd uptake in turnip plants in different studies (Page et al., 1972; Mani and Kumar, 2004). In addition, environmental conditions including medium, pH, organic matter and other ion contents could affect the absorption of turnip to Cd to different degrees (Mani and Kumar, 2004; Mani et al., 2007; Parveen et al., 2013). Therefore, experiments performed under the same environments are necessary in future studies.

As shown in **Figure 3**, the Cd concentrations in the AG parts of most landraces exceeded or approximated 100 mg kg^-1^ DW. However, it was still not rigorous to classify these landraces as Cd hyperaccumulators. Based on the current definition, Cd hyperaccumulators must satisfy a further three requirements including that both the EC and TF should greater than 1 (He, 2013). Furthermore, the plant growth should not be significantly restrained when subjected to Cd-polluted soil (He, 2013). Based on these requirements, we identified five turnip landraces (KTRG-B14, KTRG-B45c, KTRG-B50, KTRG-B56 and KTRG-B57) that may be considered Cd hyperaccumulators (**Figures 2** and **3, Table 2**). In addition, three landraces (KTRG-B13, KTRG-B48a and KTRG-48b) were expected to be classified in Cd hyperaccumulators; their biomasses were not significantly reduced under the Cd treatment but their Cd concentrations did not exceed 100 mg kg^-1^. Nonetheless, the Cd concentrations in their AG parts were expected to be more than 100 mg kg^-1^ DW under a longer growth period.

### Potential Application of Turnip in the Phytoremediation of Contaminated Soil

Phytoremediation is often considered as the most promising remediation approach of HM contaminated soil as it is a cost-effective and environmentally friendly technology (Kramer, 2005; Long et al., 2013). Phytoremediation can mainly be divided into phytoextraction and phytostabilization according to their respective principles (Ferraz et al., 2012). Plant extraction is directly dependent on plants absorbing and transferring HMs to ground parts to remove them (Pilon-Smits, 2005). The key to plant extraction is to find HM hyperaccumulators with fast growth, large biomass and high accumulation capability (Ferraz et al., 2012). According to a Chinese review article (He, 2013), more than 500 HM hyperaccumulators have been found or reported around the world, but most of them (about 300 species) were nickel hyperaccumulators. Compared with these data, the number of Cd hyperaccumulators remains limited. There were just 20–30 Cd-hyperaccumulation plants that have been reported by 2010 (He, 2013). Thus, our findings for turnip should provide great resources for phytoremediation for Cd polluted soil. Several studies have shown that some species, such as *Convolvulus arvensis* (Gardea-Torresdey et al., 2004) and *Salsola kali* (de la Rosa et al., 2004), have strong Cd accumulation capabilities but do not strictly conform to the traditional definition of a Cd hyperaccumulator, as they showed higher Cd accumulation concentrations in UG parts than in AG parts. However, these plants were still suitable for soil remediation.

Besides Cd accumulation capability, plant biomass is also a key factor that decides the Cd removal efficiency, which is supported by our results. The total Cd contents in individual plant of the five Cd-hyperaccumulation turnip landraces (KTRG-B14, KTRG-B45c, KTRG-B50, KTRG-B56, and KTRG-B57) were not the highest (**Figure 5**). Conversely, those landraces with lower Cd concentrations (e.g., KTRG-B13 and KTRG-B28) in plants enriched much larger masses of Cd (**Figures 3** and **5**). Indeed, many Cd hyperaccumulators, especially some wild plants, usually grow slowly and have smaller biomasses, which severely limits their phytoremediation efficiencies (Rungruang et al., 2011; Mani et al., 2016). Like many other cruciferous vegetables, turnip has the biological characteristics of rapid growth and high biomass and is easy to harvest. Together with our results in the present study, turnip has great potential in phytoremediation for Cd polluted soil. In addition, territory restriction is another reason for preventing the application of some Cd hyperaccumulators in environmental remediation (Wei and Chen, 2001). However, turnip has the advantage of extensive cultivation regions, even including the Tibetan Plateau. Therefore, although it has not yet been applied in practical soil remediation, turnip is a promising candidate that requires further research attention.

## Conclusion

The results of this study support previous findings that turnip is an effective Cd-accumulating species. The mean Cd concentrations in the AG and UG part were shown to reach 99.48 and 44.03 mg kg^-1^ DW, respectively, at the 30-day growth stage. The mean EC of the AG part and the mean TF was 1.99 and 2.61, respectively. However, significant intraspecific differences in Cd tolerance and accumulation capabilities were found. According to the current standard of Cd hyperaccumulators, five out of the studied 18 turnip landraces could be considered representative Cd hyperaccumulators and another three landraces have the potential to be considered Cd hyperaccumulators after further research. Further analysis showed that the total Cd content accumulated by individual plants was significantly different among turnip landraces at the studied growth stage. The results indicated that Cd removal efficiencies of turnip landraces were dependent on both the Cd accumulation capacity and biomass of plants. Based on the strong accumulation capability of Cd and biological characteristics such as their rapid growth and wide distribution, we suggest that turnip has an excellent potential for phytoremediation of Cd-contaminated soil. In addition, the findings of this study indicate that it is not advisable to consume turnips cultivated in an environment that exceeds safe Cd levels.

## Author Contributions

YpY and XL conceived and designed the experiments. YY and YpY collected the seeds. XL, XZ, BL, and YW performed the experiments. XL analyzed the data and wrote the manuscript. YpY and HS revised the manuscript.

## Conflict of Interest Statement

The authors declare that the research was conducted in the absence of any commercial or financial relationships that could be construed as a potential conflict of interest.

## Abbreviations

AG: aboveground
DW: dry weight
EC: enrichment coefficient
HM: heavy metal
TF: translocation factor
UG: underground
