# Supplementary material for: Cadmium Accumulation Characteristics in Turnip Landraces from China and Assessment of Their Phytoremediation Potential for Contaminated Soils
Source: Front Plant Sci. 2016 Dec 9;7:1862. doi: 10.3389/fpls.2016.01862 (PMC5145853; doi:10.3389/fpls.2016.01862)
Supplement: Supplementary file 1 [file Image_1.PDF]

Supplementary Material

Cadmium Accumulation Characteristics in Turnip Landraces from China and Assessment of their Phytoremediation Potential for Contaminated Soils

Xiong Li, Xiaoming Zhang, Ya Yang, Boqun Li, Yuansheng Wu, Hang Sun, Yongping Yang*

*** Correspondence:** Corresponding Author: yangyp@mail.kib.ac.cn

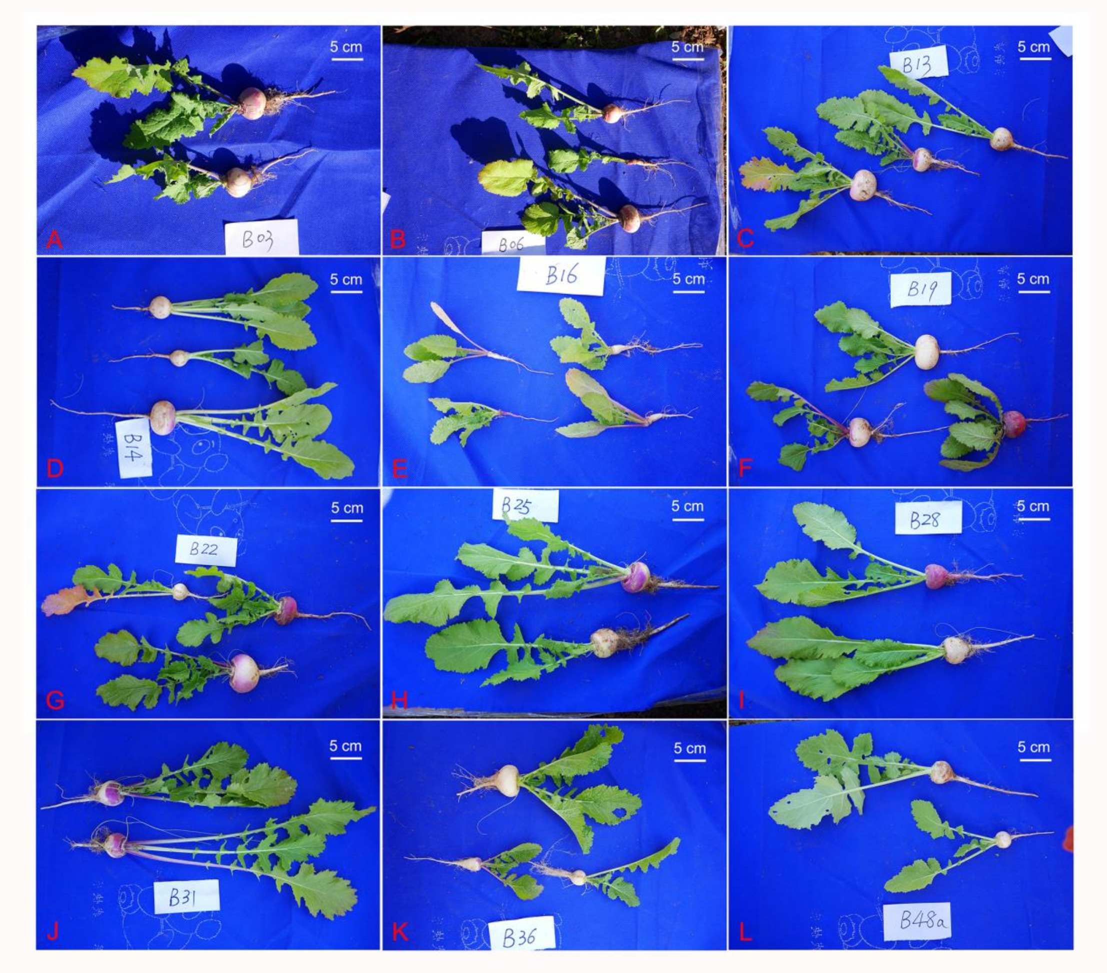

Figure S1 Morphological differences of twelve representative turnip landraces used in this study. The plants were grown under the same environmental conditions and photographed at the same time. A: KTRG-B03; B: KTRG-B06; C: KTRG-B13; D: KTRG-B14; E: KTRG-B16; F: KTRG-B19; G: KTRG-B22; H: KTRG-B25; I: KTRG-B28; J: KTRG-B31; K: KTRG-B36; L: KTRG-B48a.
